# Uncertainty analysis and optimization of laser thermal pain treatment

**DOI:** 10.1038/s41598-023-38672-y

**Published:** 2023-07-19

**Authors:** Honghua Liu, Chang She, Zhiliang Huang, Lei Wei, Qian Li, Han Peng, Mailan Liu

**Affiliations:** 1 Hunan University of Chinese Medicine, Changsha, 410208 People’s Republic of China; 2 Hunan City University, Yiyang, 413000 People’s Republic of China; 3 Hunan Institute of Science and Technology, Yueyang, 414006 People’s Republic of China

**Keywords:** Mathematics and computing, Software, Pain

## Abstract

Uncertainty in operating parameters during laser thermal pain treatment can yield unreliable results. To ensure reliability and effectiveness, we performed uncertainty analysis and optimization on these parameters. Firstly, we conducted univariate analysis to identify significant operational parameters. Next, an agent model using RBNN regression determined the relationship between these parameters, the constraint function, and the target function. Using interval uncertainty analysis, we obtained confidence distributions and established a nonlinear interval optimization model. Introducing RPDI transformed the model into a deterministic optimization approach. Solving this with a genetic algorithm yielded an optimal solution. The results demonstrate that this solution significantly enhances treatment efficacy while ensuring temperature control stability and reliability. Accounting for parameter uncertainties is crucial for achieving dependable and effective laser thermal pain treatment. These findings have important implications for advancing the clinical application of this treatment and enhancing patient outcomes.

## Introduction

### Motivation and incitement

Pain and associated abnormal sensations are often linked to current or potential tissue and nerve damage^1–3^. Proper nociceptive stimulation techniques can facilitate the early diagnosis of neurological disorders^2^, which can enable patients to receive timely treatment and prevent deterioration. Additionally, these techniques can provide a more comprehensive understanding of the pathogenesis of the disorders^4,5^, which can serve as an important theoretical basis for their eradication. The laser-evoked potential (LEP) technique is one such nociceptive stimulation technique that stimulates sensory nerve endings and induces pain by increasing the skin tissue's temperature through laser irradiation^4^. The thermal pain stimulation produced by the LEP technique conforms to the stimulation pattern of human senses and has great potential for development in nociceptive research^6,7^. Since about 90% of the infrared laser energy is absorbed by the more superficial stratum corneum and epidermis, the activation of nociceptive receptors is mainly achieved by thermal conduction. Therefore, the temperature of nociceptive receptors is used as a target parameter for laser thermal therapy for pain. If the temperature of the nociceptive receptors is too low, it can be difficult to achieve the conditions necessary for their activation. Conversely, if the temperature is too high, it may result in damage to sensory nerve endings or generate false-positive signals, which can impact the accuracy of the study results. Therefore, the intensity of the stimulus, i.e., the temperature of the nociceptive receptors, must be controlled between the nociceptive and tissue damage thresholds.

A comprehensive understanding of the challenges in this field can be gained by analyzing the temperature distribution of the medium. Ding H. et al.^8^ simulated and compared the temperature distribution induced by continuous and pulsed lasers on human skin. To account for the diverse thermal characteristics of distinct skin layers, including the stratum corneum, living epidermis, and dermis, scholars have introduced two-dimensional and three-dimensional models derived from one-dimensional models^9–12^. Rossi F et al.^13^ developed a two-dimensional model of facial skin using the finite element method (FEM), considering different skin structures and their optical and thermal parameters. Shurrab K.M. et al.^14^ developed a three-dimensional finite element thermal model and used FEM to calculate the temperature distribution of skin tissue.

However, these studies mainly focused on modeling tissue temperature distribution with high accuracy and rarely considered the effects of different factors on nociceptive receptors. In typical laser thermal pain stimulation studies, there is significant uncertainty in several parameters, such as the laser wavelength, spot diameter, irradiation time, laser power density, ambient temperature, optical parameters of biological tissues, and thermal physical parameters. These uncertainties can have an error effect on the treatment results. To ensure objective and accurate determination of laser output dose, safe and effective evocation of nociceptive potentials, and real and reliable nociceptive potential signals by LEPs technology, it is necessary to consider the uncertainties of these operating parameters. To achieve precise and reliable measurement of laser output dose, safe and effective activation of nociceptive potentials, and acquisition of authentic and dependable nociceptive potential signals using LEP technology, it is imperative to develop an optimization method that takes into account the uncertainties associated with these operational parameters.

### Contributions and salient features

In recent years, various optimization algorithms and neural networks have been widely applied in the fields of optimization problems and pattern recognition. Optimization algorithms, such as variants of Chaotic Grey Wolf Heuristic, Marine Predator Optimization Using the Key Term Separation Technique, Dwarf Mongoose Optimization Metaheuristics, Design of Aquila Optimization Heuristic and Design of Nonlinear Marine Predator Heuristics, have demonstrated their effectiveness in solving complex optimizatio Zn problems by mimicking natural or computational processes^15–19^. Neural networks have gained significant attention for their ability to learn complex patterns and make predictions based on vast amounts of data^20–25^. Some current research in the medical field has focused on numerical simulations and computational approaches for understanding complex phenomena^26^. Researchers, such as Mubashir et al.^27^, have explored the generation of traveling wave solutions using the He-Laplace algorithm. Additionally, Al Alwan et al.^28^ have investigated the formation of exact solitary waves in the generalized Calogero-Bogoyavlenskii-Schiff equation. Furthermore, Partohaghighi et al.^29^ have analyzed fractional differential equations using different methods. Shaikh et al.^30^ have proposed a nonlinear structure model for chemical reactions, emphasizing the significance of existence and uniqueness through numerical modeling. These studies contribute to the expanding knowledge in their respective areas of research.

In this paper, we propose an effective optimization method for uncertainty laser thermal pain stimulation. By employing RBNN as a surrogate model, we train it to predict the output response for new input variables using a limited number of simulation runs. This approach improves computational efficiency by reducing the need for repetitive simulations. The RBNN model serves as a reliable proxy, enabling accurate predictions based on the given input variables. Interval optimization is a new uncertain optimization method that fully considers the fluctuation range of the operating parameters^31,32^. Interval optimization has gained increasing attention in the fields of mechanics, acoustics, and heat transfer^33–38^. By fully considering the influence of the uncertainty of the operating and skin parameters on the constraints, interval optimization ensures that the obtained optimal solution can meet the reliability requirements. In this paper, we use the interval optimization method as an uncertainty optimization method to develop an optimization model for the gentle moxibustion treatment process.

### Paper organization

**Figure 1:**
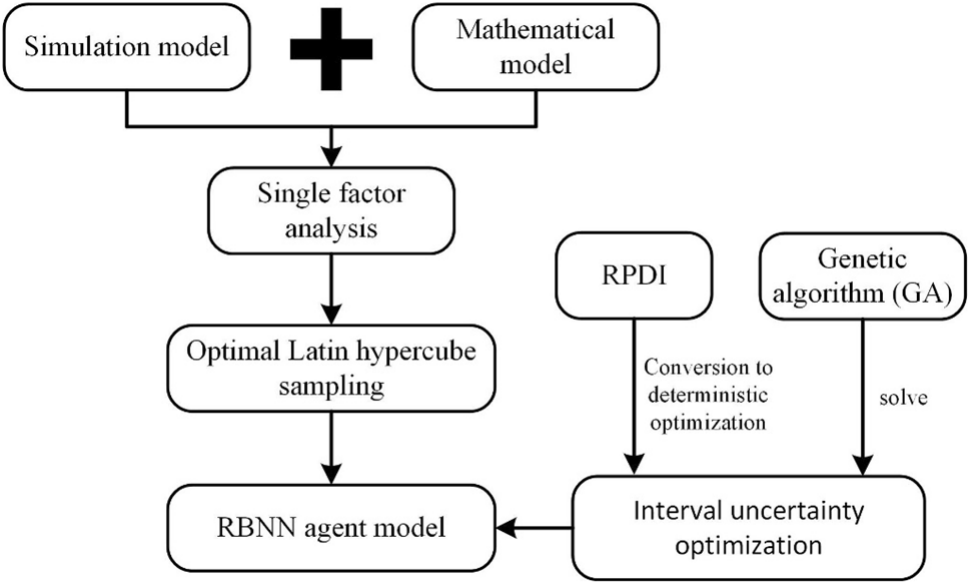
The graphical representation of the study.

In the method section, we initially developed a simulation model and mathematical model for laser thermal pain treatment. Then, we conducted a single-factor analysis of the operating parameters. Subsequently, we established an RBNN agent model. Afterwards, we utilized the interval uncertainty analysis method to construct a nonlinear interval optimization model. This model was converted into a deterministic optimization model by incorporating RPDI. Finally, we employed a genetic algorithm to solve the deterministic problem. In the results and discussion section, we first analyzed the influence of the operating parameters by integrating the outcomes of the single-factor analysis and orthogonal experimental analysis. Next, we assessed the error of the RBNN model. Additionally, we performed uncertainty analysis on the operating parameters and achieved nonlinear uncertainty optimization of the target parameters. The conclusion section provides a summary of the findings. We have created a graphical representation of the study to effectively capture the key aspects of our research (Fig. 1).

## Method

### Simulation model building

In this paper, we established a 3D physical simulation model of laser thermal pain treatment using COMSOL to study the relationship between the temperature within the skin tissue and the operating parameters. Human skin is composed of three layers, namely the epidermis, dermis, and subcutaneous tissues. Due to the short duration of laser irradiation, the skin tissues' temperature rise is limited, so the modeling does not include subcutaneous tissues, such as fat and muscle. The two remaining layers of biological tissue are refined into five parts, including the stratum corneum, epidermis, upper dermis, blood layer, and subdermal, with thicknesses of 10 μm, 80 μm, 260 μm, 150 μm, and 1500 μm, respectively.

**Figure 2:**
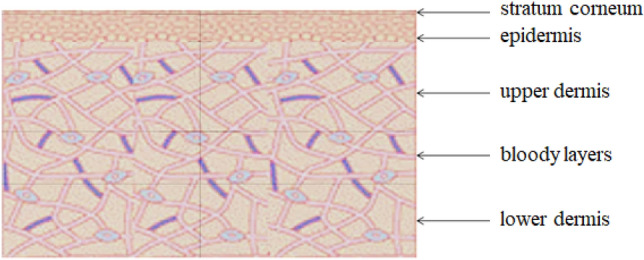
The biological tissue model.

The biological tissue model is shown in Fig. 2, and the specific properties are listed in Table 1^9^.

**Table 1:** Biological tissue model.

biological tissue	$C_{p}$(${\text{J}} \cdot {\text{kg}}^{ - 1} \cdot {\text{K}}^{ - 1}$)	${\uprho }$(${\text{kg}} \cdot {\text{m}}^{ - 3}$)	*k* ${\text{(W}} \cdot {\text{m}}^{ - 1} \cdot {\text{K}}^{ - 1} {)}$
Epidermis	2250	1210	0.197
Upper dermis	3350	1090	0.422
Blood layer	3670	1060	0.486
Lower dermis	3350	1090	0.422

**Figure 3:**
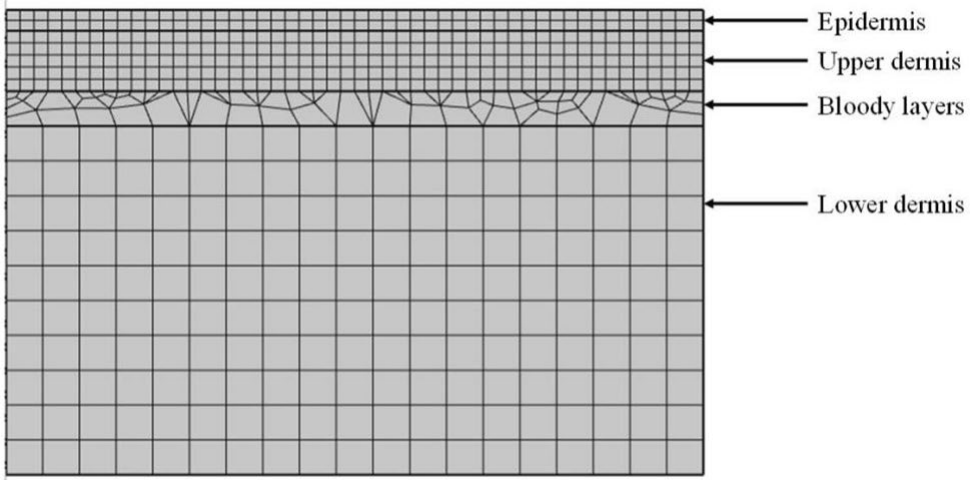
The grid structure of biological tissue.

This paper replaces the cuticle with the epidermis when building the physical model. The triangular mesh divides the axisymmetric model in this paper, as shown in Fig. 3. In this model, the maximum size of the grid cell is 159 μm, and the minimum size is 0.9 μm.

By employing an appropriate mesh division, a more precise representation of temperature changes in both the skin surface and deeper tissues can be achieved, resulting in simulation results of higher accuracy. Additionally, this approach allows for optimization between precision and computational resource usage.

### Mathematical modeling

The laser transmits heat to the skin through radiation. The heat absorbed in the skin through thermal radiation becomes the biological group's heat source. It is the basis for heat transfer analysis in biological tissues and leads to temperature field distribution throughout the tissue being treated. The heat transfer in biological tissues can be solved by the Pennes equation^39^.

1 $$\rho c\frac{\partial T}{{\partial t}} = \nabla \cdot \left( {k\nabla T} \right) + \omega_{b} C_{b} \left( {T_{b} - T} \right) + q_{m} + q_{r}$$

Here, T, ρ, c, and k are the temperature, density, specific heat, and thermal conductivity of the biological tissue, respectively. $\omega_{b}$ denotes the blood perfusion. ${\text{C}}_{\text{b}}$, ${\text{T}}_{\text{b}}$ represent the specific heat and temperature of blood. ${\text{q}}_{\text{m}}$ is the metabolic heat production rate, and ${\text{q}}_{\text{r}}$ is the external heat source.

The laser beam profile is modeled as a Gaussian distribution with a standard deviation of 2.85 mm. It was fitted by the laser system model and corresponded to a 1/e two diameter of 11.4 mm. Light absorption in tissue was modeled as exponential decay using the Beer-Lamberts law. Thus, Q is formulated as the formula.

2 $$Q\left( {r,t} \right) = P_{in} \mu_{\alpha } e^{{\left( { - \mu_{\alpha } ,z} \right)}} \frac{1}{{\sigma \sqrt {2\pi } }}e^{{\left( {\frac{{ - r^{2} }}{{2\sigma^{2} }}} \right)}}$$

Here, ${\text{P}}_{\text{in}}$ represents the laser power. $\mu_{\alpha }$ is the absorption coefficient of the tissue. $\mu_{s}$ is the scattering coefficient of the tissue. *z* denotes the depth from the tissue surface. $\sigma$ is the spot radius. r is the distance to the beam center. Table 2 shows the optical parameters of the biological tissue model.

**Table 2:** Biological tissue optical parameters^34^.

biological tissue	$\mu_{\alpha } \left( {{\text{cm}}^{ - 1} } \right)$	$\mu_{s} \left( {{\text{cm}}^{ - 1} } \right)$
Epidermis	2.7	450
Dermis	0.7	180
Blood	0.5	44.9

In laser thermal pain stimulation treatment, it is important to consider the optimal temperature range that will result in realistic and reliable nociceptive potential signals to the skin tissue nociceptors, while avoiding discomfort or irreversible damage to the skin tissue. Previous research by Tillman et al. found that the average depth of C nociceptive fibers in monkeys' hairy skin was 201 μm. In human hairy skin, the "Aδ" fibers penetrate the dermis to the epidermis. Hairless skin typically has fewer nociceptors than hairy skin. Previous studies have examined laser-evoked responses, such as latency and pain intensity, in hairless and hairy skin of type I and type II fiber groups^40–42^. The response thresholds for type I and type II were found to be 52 °C and 46 °C, respectively.

Clinical experience shows that when the human tissue temperature ranges from 37 °C to 43 °C, only a warm sensation is observed, with no significant effect or damage to the tissue for a longer duration. When the tissue temperature ranges from 43 °C to 52 °C, short-time irradiation can activate the injury receptors without causing permanent damage to the tissue. However, when the tissue temperature reaches 60 °C, short-time irradiation can denature the proteins and collagen in the tissue, resulting in cellular damage and tissue coagulation. Therefore, this paper takes 52 °C as the tissue damage threshold, where skin surface temperature above this level could be detrimental to the patient. Nociceptive receptors in the skin are distributed within the range of 20 μm to 570 μm^43^. Thus, to generate a more realistic and reliable nociceptive potential signal, it is necessary to achieve thermal skin penetration, i.e., the maximum temperature at 20 μm below the skin surface, with skin surface temperatures below 52 °C. Therefore, in this paper, skin surface temperature and thermal skin penetration are used as indicators of laser thermal pain stimulation treatment.

### The setting of working parameters

The temperature distribution of laser-irradiated skin tissue is primarily affected by laser power, spot radius, irradiation time, and ambient temperature. To investigate the effects of these individual parameters on thermal analgesia treatment, we employed a numerical model. Specifically, we adjusted one parameter while keeping the other three parameters fixed. The parameter values for each level are presented in Table 3.

**Table 3:** The parameter values at various levels.

Levels	Laser Power (W)	Spot Radius (mm)	irradiation time (s)	ambient temperature (°C)
1	5	1	0.2	10
2	7.5	1.5	0.4	25
3	10	2	0.6	35

### Single factor analysis of laser thermal pain stimulation

One-factor analysis involves analyzing only one variable that is experimentally treated in a single direction. In the context of laser thermal pain stimulation, single-factor analysis enables the exploration of the influence of different factors on the index parameters and the identification of factors with significant effects. In practical applications, the factors that affect the skin tissue's temperature distribution after laser irradiation are mainly external factors, which include laser power, spot radius, irradiation time, and ambient temperature. In this paper, we conducted single-factor analysis on these external factors by setting up external factors and designing simulations.

### Establishment of a surrogate model

In laser thermal pain treatment, the effect indicators are mainly thermal penetration HPM and skin surface temperature ST. Four optimization factors, such as laser power, spot radius, irradiation time, and ambient temperature, were selected to study the relationship with the effect indicators of laser thermal pain stimulation.

### Optimal Latin hypercube sampling

**Figure 4:**
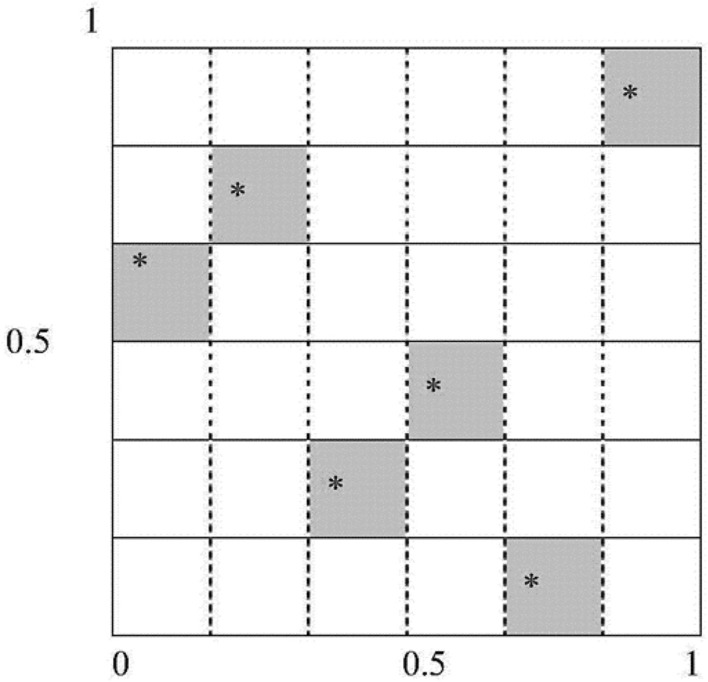
The Latin hypercube sampling.

**Figure 5:**
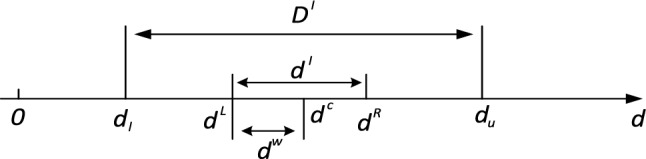
The relationship between the interval and the design range.

Interval uncertainty optimization aims to find the optimal design that meets the reliability requirements and minimizes (or maximizes) the value of the objective function. Due to the time-consuming finite element analysis, it is computationally expensive to invoke the finite element model for the optimization solution directly. A radial basis neural network (RBNN) considering the optimization parameters is established for the constraint and objective functions to improve efficiency. The approximate response function is given to solve the interval uncertainty optimization problem. Experimental design is required to build the RBNN. Latin hypercube sampling is a widely used multidimensional hierarchical experimental design method^44,45^. The sampling steps are as follows. ① Each space in the n-dimensional space is divided into m intervals according to equal probability. ② Each interval is randomly sampled once to ensure that each dimension is studied once. ③The number of samples is randomly paired to generate the type matrix. The results are shown in Fig. 4. In this paper, the Latin hypercube is used to sample the design points, which is beneficial to the uniform distribution of sampling. In this study, 50 experimental design points were obtained using the optimal Latin hypercube sampling method within the design range of the operational parameters (Fig. 5).

### RBNN training

Based on the results of the sampling points, an agent model is developed, and the response surface of the entire design domain is obtained. In this study, RBNN model regression was used to develop the proxy model of the objective function^46,47^. The objective parameters of any design point in the entire design space can be obtained with the accuracy of the proxy model based on the established parameters. The operational parameter data are first normalized. Since different operational parameters have different magnitudes and magnitude units, such a situation can affect the results of data analysis, so the data are normalized in order to eliminate the influence of magnitudes between operational parameters. The relative error (RE) between the simulation results y(x) and the approximation of the neural network function f(x) can be expressed by Eq. (3)^48^

3 $$RE = \frac{{y(x) - \overset{\lower0.5em\hbox{$\smash{\scriptscriptstyle\frown}$}}{y} (x)}}{y(x)}$$

Also, the accuracy of the developed proxy model is evaluated through the root mean square error (RMSE), which can be expressed as Eq. (4–7)

4 $$RMSE = \sqrt{\frac{SSE}{k}}$$

5 $$R^{2} = 1 - \frac{SSE}{{SST}}$$

6 $$SSE = \sum\limits_{i = 1}^{k} {(y_{i} - \overset{\lower0.5em\hbox{$\smash{\scriptscriptstyle\frown}$}}{y}_{i} )^{2} }$$

7 $$SST = \sum\limits_{i = 1}^{k} {(y_{i} - \overline{y})^{2} }$$

Here, SSE is the sum of squared errors, k is the number of samples (k = 50), and SST denotes the total sum of squares. Is the response value of the simulation model at the i-th sample, $$\overset{\lower0.5em\hbox{$\smash{\scriptscriptstyle\frown}$}}{y}_{i}$$ is the response value of the surrogate model at the i-th sample, and $$\overline{y}$$ represents the mean of $$y_{i}$$.

### Objective function uncertainty analysis

When nociceptive potentials are induced using laser radiation to the skin tissue, some of the energy is absorbed by the skin, and heat is generated by absorption and scattering of the skin tissue, enough heat to activate the nociceptive receptors. Various factors such as laser power, spot radius, irradiation time, and ambient temperature can influence the temperature of the laser-irradiated skin, and there is also uncertainty in these parameters, which fluctuate in an interval range, making it difficult to guarantee that each treatment is performed under the same conditions, leading to large temperature fluctuations that may not meet treatment expectations or even damage the skin tissue. Therefore, the optimal solution given by the deterministic optimal design only satisfies the constraint when the working parameter is a definite value. This so-called optimal solution cannot really have fluctuations in the working parameters, which, as mentioned before, is unreliable during the actual treatment. Therefore, in order to obtain a stable optimal solution for the laser thermal pain stimulation process, it is necessary to quantify the operating parameters as uncertain parameters and also to develop an optimal design of uncertain parameters. In this paper, the uncertainty of each operating parameter is expressed as an interval. Using as the design operating parameter, the range of variation of this operating parameter can be described as

8 $$d \in d^{I} = \left[ {d^{L} ,d^{R} } \right]$$

Here, $d^{I}$ represents the parameter interval, $d^{L}$ and $d^{R}$ are the lower and upper bounds of the parameter interval, respectively. The interval is also denoted as Eq. (9)^49,50^ :

9 $$\begin{aligned} d \in d^{I} = & \left[ {d^{L} ,d^{R} } \right] = \{ d\left| {d^{L} \le d \le } \right.d^{R} ,d \in R\} \\ = & \{ d\left| {d^{c} - d^{\omega } \le d \le } \right.d^{c} + d^{\omega } ,d \in R\} \\ = & \left\langle {d^{c} ,d^{\omega } } \right\rangle ,{\kern 1pt} {\kern 1pt} {\kern 1pt} i = 1,2,3 \\ \end{aligned}$$

Here, $d^{c}$ and $d^{\omega }$ represent the midpoint and radius of the interval, as Eq. (10).

10 $$d^{c} = \frac{{d^{L} { + }{\kern 1pt} {\kern 1pt} {\kern 1pt} d^{R} }}{2}{\kern 1pt} {\kern 1pt} {\kern 1pt} ,{\kern 1pt} {\kern 1pt} {\kern 1pt} {\kern 1pt} d^{\omega } = \frac{{d^{R} { - }{\kern 1pt} {\kern 1pt} {\kern 1pt} d^{L} }}{2}$$

As shown in the equation, the interval can be determined by the midpoint $d^{c}$ and the radius $d^{\omega }$. The midpoint is usually taken as the middle value of the parameter, and the radius reflects the degree of variation of the operating parameter.$d^{I}$ should be chosen within a reasonable design domain in the interval uncertainty optimization. The design domain of uncertain parameters is denoted as $D^{I}$, and its lower and upper bounds are $d_{l}$ and $d_{u}$, respectively.

### Optimal design of nonlinear intervals for laser thermal pain stimulation

Due to the need for interval optimization of thermal penetration under uncertainty, the maximum thermal penetration is achieved while keeping the working parameters as much as possible under the skin surface temperature constraint. The interval optimization problem can be expressed as Eq. (11)^51^:

11 $$\begin{gathered} \mathop {\min }\limits_{{X^{I} }} H\left( {X^{I} } \right) \hfill \\ \;\;\;\;s.t. \hfill \\ \;\;\;\;S_{j} \left( {X^{I} } \right) \le b_{j} {\kern 1pt} {\kern 1pt} ,{\kern 1pt} {\kern 1pt} {\kern 1pt} {\kern 1pt} j = 1,2, \cdot \cdot \cdot ,l \hfill \\ \;\;\;\;X_{l} \le X^{I} \le X_{u} \hfill \\ \;\;\;\;X_{i}^{I} = \left[ {X_{i}^{L} ,X_{i}^{R} } \right]{\kern 1pt} {\kern 1pt} {\kern 1pt} i = 1,2, \cdot \cdot \cdot ,n \hfill \\ \end{gathered}$$

Here, $X^{I} = \left[ {X_{1}^{I} ,X_{2}^{I} \cdot \cdot \cdot X_{n}^{I} } \right]$ is an n-dimensional interval design vector, $X_{l}$ and $X_{u}$ are the lower and upper bounds of the interval variable. $H$ and $S$ represent the objective and constraint functions, $b_{j}$ denotes the allowable value of the j-th constraint, l represents the number of constraints. The superscripts I, L, and R denote the parameter interval and its lower and upper bounds, respectively. Therefore, the interval uncertainty optimization of laser thermal pain stimulation is performed to obtain the optimal operating parameter interval. By optimizing the operating parameters, the optimal solution of the objective function is achieved while ensuring reliability.

To construct an interval optimization model for the laser thermal pain stimulation treatment process, the target and constraint parameters should be set first. According to the above discussion, in this paper, thermal penetration is set as the target parameter, and skin surface temperature is set as the constraint function. Due to the uncertainty of the operating parameters, both thermal penetration and skin surface temperature are variable and uncertain values within the interval. The interval design variables contain the operating parameters such as laser power, spot radius, irradiation time, and ambient temperature, as shown in Table 3 above. The objective function and constraint function values can be optimized by changing the operating parameters during treatment.

Based on the above discussion, the interval optimization model of the laser thermal pain stimulation treatment process can be formulated as follows:

12 $$\begin{gathered} \mathop {\max }\limits_{{d^{c} ,d^{\omega } }} H\left( {d^{I} } \right) \hfill \\ \;\;\;\;s.t. \hfill \\ \;\;\;\;S\left( {d^{I} } \right) \le b \hfill \\ \;\;\;\;d_{l} \le d^{I} \le d_{u} \hfill \\ \;\;\;\;d_{i}^{I} = \left[ {d_{i}^{L} ,d_{i}^{R} } \right]{\kern 1pt} {\kern 1pt} {\kern 1pt} i = 1,2,3 \hfill \\ \end{gathered}$$

Here, E Indicates thermal penetration of laser thermal pain treatment, F denotes the ambient temperature constraint function, both of which are functions on the working parameters and are influenced by the working parameters; $d^{I} { = }\left[ {d_{1}^{I} {\kern 1pt} {\kern 1pt} d_{2}^{I} \cdot \cdot \cdot d_{n}^{I} } \right]$ is an n-dimensional interval design vector; b is the value of the desired temperature, i.e., 52 °C. $d_{l}$ and $d_{u}$ represent the lower and upper bounds of the design domain. $d_{l}^{L}$ and $d_{l}^{R}$ denote the lower and upper bounds of each parameter interval. $d_{i} {\kern 1pt} {\kern 1pt} {\kern 1pt} {\kern 1pt} {\kern 1pt} {\kern 1pt} i = 1,2,3$ are the moxibustion parameter intervals, written as Eq. (13).

13 $$d_{i}^{I} = \left\langle {d_{i}^{c} ,d_{i}^{\omega } } \right\rangle ,{\kern 1pt} {\kern 1pt} {\kern 1pt} i = 1,2,3$$

Thus, the laser thermal pain treatment process interval model can be expressed as Eq. (14)

14 $$\begin{gathered} \mathop {\max }\limits_{{d^{I} }} H\left( {\left\langle {d_{i}^{c} ,d_{i}^{\omega } } \right\rangle } \right) \hfill \\ \;\;\;\;s.t. \hfill \\ \;\;\;\;S\left( {\left\langle {d_{i}^{c} ,d_{i}^{\omega } } \right\rangle } \right) \le b \hfill \\ \;\;\;\;d_{l} \le \left\langle {d_{i}^{c} ,d_{i}^{\omega } } \right\rangle \le d_{u} \hfill \\ \end{gathered}$$

Here, $d^{c} { = }\left[ {d_{1}^{c} {\kern 1pt} {\kern 1pt} d_{2}^{c} \cdot \cdot \cdot d_{n}^{c} } \right]$ is the n-dimensional vector of the interval parameter midpoints. $d^{w} { = }\left[ {d_{1}^{w} {\kern 1pt} {\kern 1pt} d_{2}^{w} \cdot \cdot \cdot d_{n}^{w} } \right]$ is an n-dimensional vector of the interval parameter radius.

Before solving the interval optimization design model, the critical concept of reliability-based interval likelihood degree (RPDI) is proposed to be useful for further simplifying the solution process.

For two different intervals $A^{I}$ and $B^{I}$, RPDI can be expressed as Eq. (15)^52^:

15 $$p_{r} \left( {A^{I} \le B^{I} } \right) = \frac{{B^{R} - A^{L} }}{{2A^{\omega } { + }2B^{\omega } }}$$

Here, $p_{r}$ represents the similarity of the intervals. $p_{r} \left( {A^{I} \le B^{I} } \right)$ has the following features^52^:

① $${ - }\infty \le p_{r} \left( {A^{I} \le B^{I} } \right) \le { + }\infty$$;
② if $$A^{R} \le B^{L}$$, $$p_{r} \left( {A^{I} \le B^{I} } \right) \ge 1$$;
③ if $$A^{L} \le B^{R}$$, $$p_{r} \left( {A^{I} \le B^{I} } \right) \le 0$$;
④ if $$p_{r} \left( {A^{I} \le B^{I} } \right){ = }q$$, $$p_{r} \left( {B^{I} \le A^{I} } \right){ = }1 - q$$ where $$q \in \left( { - \infty , + \infty } \right)$$。

Here, $B^{I}$ degenerates into an actual number B, and the RPDI can be formulated as Eq. (16).

16 $$p_{r} \left( {A^{I} \le B^{I} } \right){ = }\frac{{B{ - }A^{L} }}{{2A^{w} }}$$

#### Solution of the nonlinear interval optimization

The constraint function is expressed as Eq. (17)^52^.

17 $$F\left( {\left\langle {d^{c} ,d^{\omega } } \right\rangle } \right) = \left[ {F^{L} ,F^{R} } \right] = \left[ {\mathop {\min }\limits_{{d \in \left( {d^{c} ,d^{\omega } } \right)}} F\left( d \right),\mathop {\max }\limits_{{d \in \left( {d^{c} ,d^{\omega } } \right)}} F\left( d \right)} \right]$$

Therefore, the constraint function is transformed into the deterministic constraint as Eq. (18):

18 $$p_{r} \left( {F\left( {\left\langle {d^{c} ,d^{\omega } } \right\rangle } \right) \le b} \right) = \frac{{b - F^{L} }}{{2F^{\omega } }} \ge \lambda$$

Here, $\lambda$ represents the interval-constrained RPDI level, which is set according to the actual usage requirements of laser thermal pain treatment. Based on the RPDI, the optimization model for laser thermal pain treatment interval design can be converted into a defined optimization problem, as Eq. (19)^50^:

19 $$\begin{gathered} \max E\left( {\left\langle {d^{c} ,d^{\omega } } \right\rangle } \right) \hfill \\ \;\;\;\;\;s.t. \hfill \\ \;\;\;\;\;p_{r} \left( {F\left( {\left\langle {d^{c} ,d^{\omega } } \right\rangle } \right) \le b} \right) = \frac{{b - F^{L} }}{{2F^{\omega } }} \ge \lambda \hfill \\ \;\;\;\;\;d_{l} \le d^{c} - d^{\omega } \le d^{c} + d^{\omega } \le d_{u} \hfill \\ \end{gathered}$$

Here, $F^{L}$ is the lower bound of thermal penetration, and $F^{w}$ represents its radius. By introducing RPDI, classical optimization algorithms can be used for solving uncertain interval optimization problems. The interval optimization model for the laser thermal pain treatment process is a nested optimization model. The optimization is obtained using the outer layer, and the inner layer is used for solving the constraint function. The outer layer of the model is optimized using a genetic algorithm (GA), and the inner layer is optimized using sequential quadratic programming (SQP).

## Results and discussion

### Single factor analysis of laser thermal pain treatment

A single-factor analysis was conducted to assess the influence of operating parameters, namely laser power, spot radius, irradiation time, and ambient temperature, on temperature distribution during laser thermal pain treatment.

### Influence of the laser power on the temperature distribution

**Figure 6:**
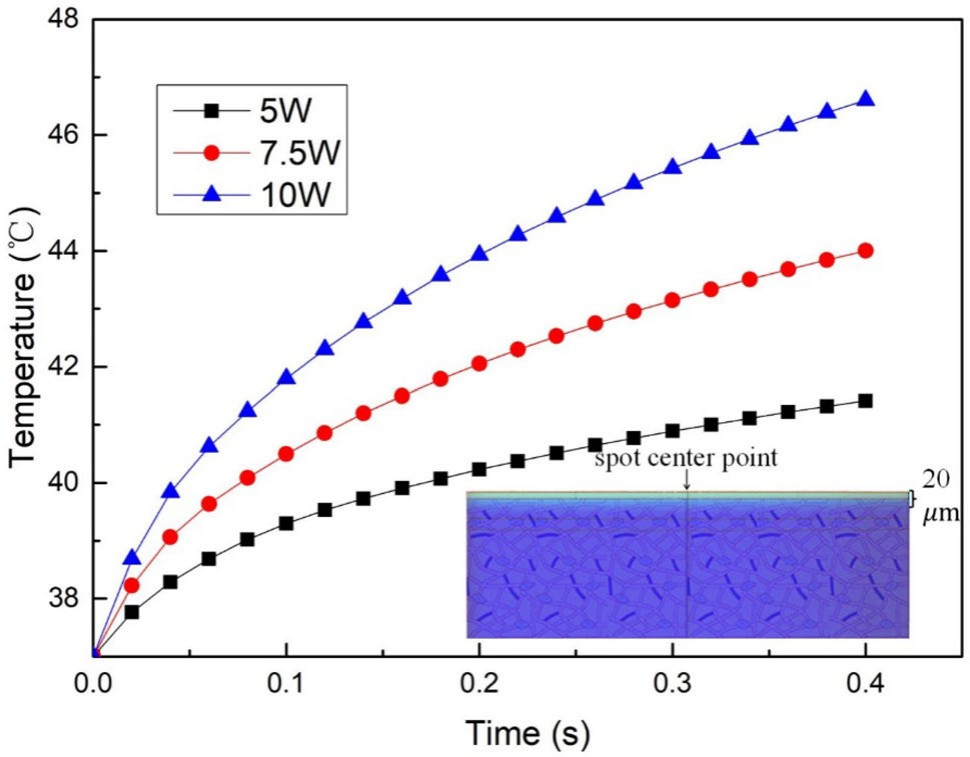
The temperature rise curve at the target point under different laser power.

The laser power was changed by fixing the spot radius, irradiation time, and ambient temperature. The temperature rise curve at the target point of biological tissue after laser irradiation was obtained, as shown in Fig. 6.

As seen in Fig. 6, an increase in laser power leads to a higher energy delivery to the next layer within the biological tissue model, resulting in a deeper temperature penetration. The plot indicates that laser power has a substantial impact on the temperature rise of biological tissues, showing a roughly linear relationship between the two variables. Specifically, increasing the laser power by 2.5W raises the temperature by approximately 3 °C.

### Influence of the spot radius on the temperature distribution

**Figure 7:**
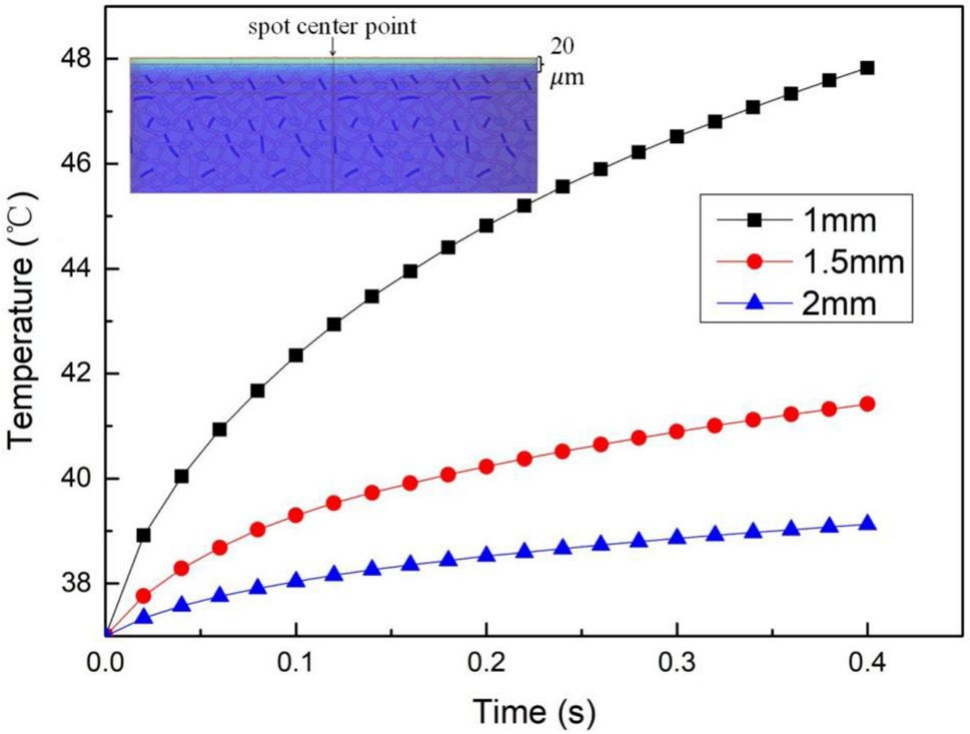
The temperature rise curve at the target point under different spot radius.

The second step is to change the spot radius. In this process, we need to fix the laser power, irradiation time, and ambient temperature. The temperature rise curve at the target point of biological tissue after laser irradiation was obtained, as shown in Fig. 7.

Figure 7 shows that the spot radius has a significant impact on the temperature change during laser irradiation. Increasing the spot radius leads to a smaller temperature change. Specifically, as the spot radius increases, the temperature rise becomes slower and the maximum temperature is lower. For instance, when the spot radius is increased from 1 mm to 1.5 mm, the temperature at the target point drops by about 6 °C. However, when the spot radius is increased from 1.5 mm to 2 mm, the temperature drop is only about 2 °C. Notably, when the spot radius reaches 2 mm, the temperature at the target point does not meet the minimum requirements for the nociceptors to generate electrical signals.

### Influence of the irradiation time on the temperature distribution

**Figure 8:**
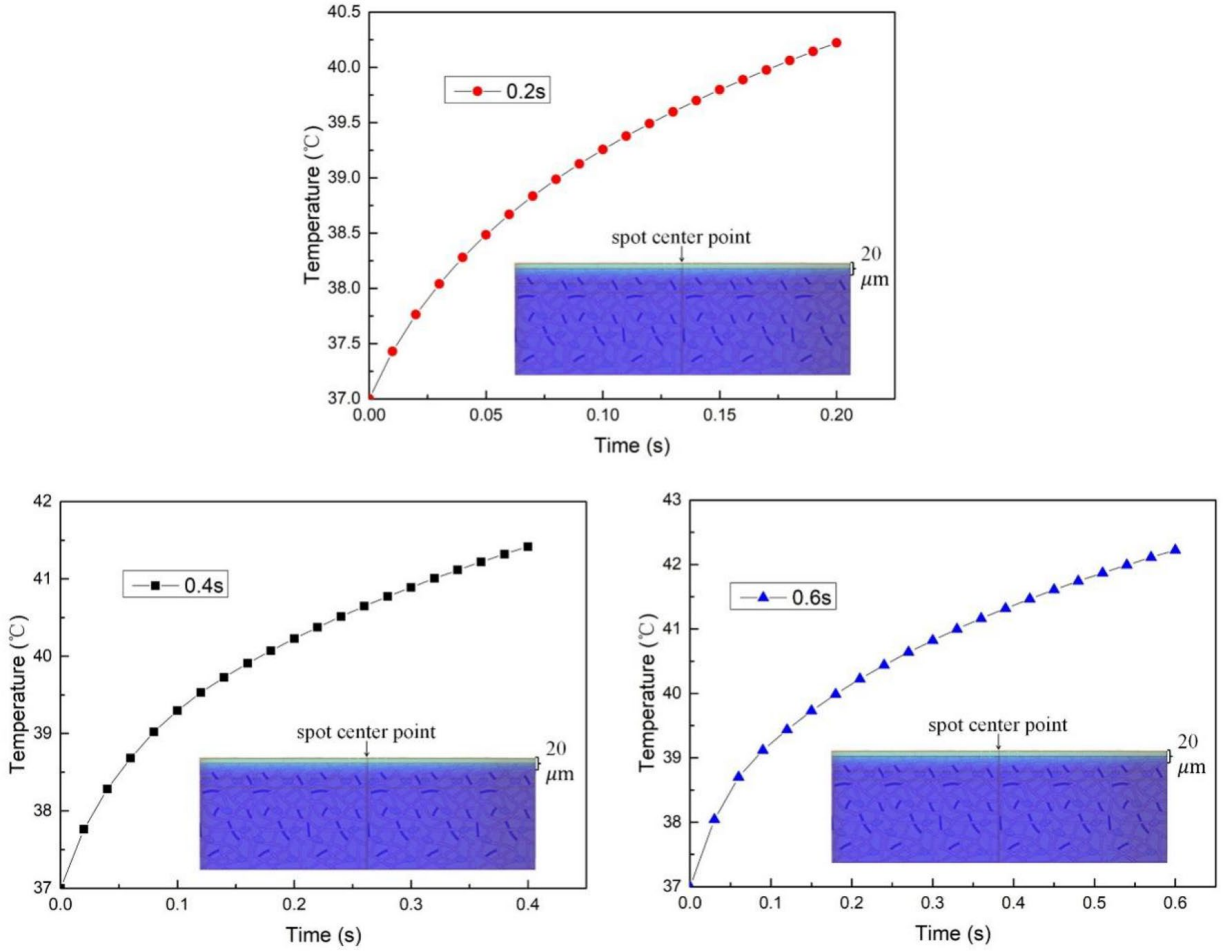
The temperature rise curves of target points under different irradiation times.

The irradiation time was changed by fixing the laser power, spot radius, and ambient temperature. The temperature rise curve at the target point of biological tissue after laser irradiation was obtained, as shown in Fig. 8.

Figure 8 shows that the duration of irradiation has a minimal effect on the temperature distribution of biological tissue during laser treatment. With an increase in irradiation time of 0.2 s, the temperature at the target point only increases by approximately 1 °C. Additionally, the shape of the temperature rise curve remains relatively stable over this short period.

### Influence of the ambient temperature on the temperature distribution

**Figure 9:**
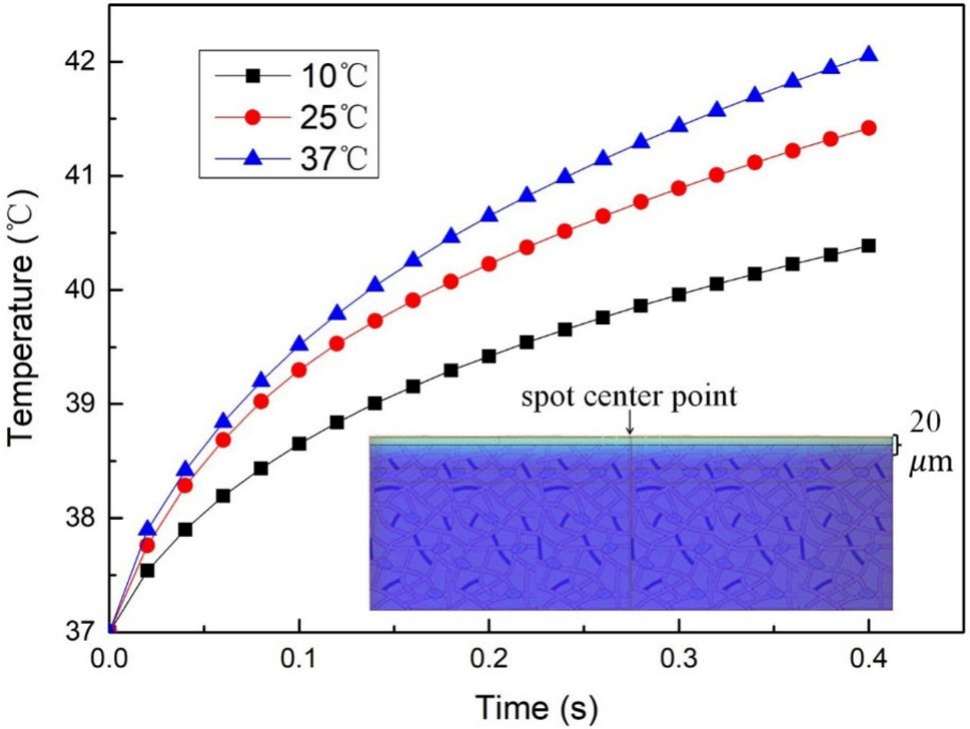
The temperature rise curve at the target point under different ambient temperature.

The ambient temperature was changed by fixing the laser power, spot radius, and irradiation time. The temperature rise curve at the target point of biological tissue after laser irradiation was obtained, as shown in Fig. 9.

Figure 9 illustrates that the impact of ambient temperature on the temperature distribution of biological tissue during laser irradiation is less significant compared to changes in laser power and spot radius.

Based on the results of the single-factor analysis, it is evident that variations in laser power, spot radius, irradiation time, and ambient temperature have notable effects on both skin surface temperature and subcutaneous 20 μm temperature. Thus, these four parameters were selected as optimization parameters to investigate their effects on the therapeutic effect of laser thermal pain.

### Orthogonal test analysis

After the single-factor analysis we obtained the simulation results under different conditions, and roughly obtained the influence of different influencing factors on the temperature of the two target points. In this part of the discussion, we will analyze the significant influencing factors and try to find the optimal parameter combination The laser power, spot radius, irradiation time, and ambient temperature were selected as the factors of the orthogonal experiment, expressed as A, B, C, and D. The number of factor levels is usually 2–4. We selected three levels for each factor, as listed in Table 4.

**Table 4:** The level of each factor.

Levels	Laser power(A) (W)	Spot radius(B)	Irradiation time(C) (s)	Ambient temperature(D) (°C)
1	5	1	0.2	10
2	7.5	1.5	0.4	25
3	10	2	0.6	35

In laser irradiation, three levels for each of the four factors were selected, written as ${\text{L}}_{9}\text{(}{3}^{4}\text{)}$.

### Analysis of orthogonal experiment results

Based on the laser irradiation simulation model, the simulation analysis was performed on the nine groups of parameters in Table 5. The temperature at the target point 20 μm below the skin surface was used to compare the results, as listed in Table 6.

**Table 5:** The standard Orthogonal Table of ${\text{L}}_{9}\text{(}{3}^{4}\text{)}$.

Experimental no	Laser power(A)	Spot radius(B)	Irradiation time(C)	Ambient temperature(D)
1	5	1	0.2	10
2	5	1.5	0.4	25
3	5	2	0.6	35
4	7.5	1	0.4	35
5	7.5	1.5	0.6	10
6	7.5	2	0.2	25
7	10	1	0.6	25
8	10	1.5	0.2	35
9	10	2	0.4	10

**Table 6:** The orthogonal experimental result.

Experimental no	Laser power(A)	Spot radius(B)	Irradiation time(C)	Ambient temperature(D)	Target point 20 μm below skin surface
1	5	1	0.2	10	44.190
2	5	1.5	0.4	25	41.418
3	5	2	0.6	35	40.320
4	7.5	1	0.4	35	54.258
5	7.5	1.5	0.6	10	44.122
6	7.5	2	0.2	25	39.628
7	10	1	0.6	25	63.521
8	10	1.5	0.2	35	44.511
9	10	2	0.4	10	41.048

### Range analysis

The range analysis (i.e., R-method) can be used to analyze the orthogonal experiment results for obtaining the optimal level combination and the influence of each factor on the experimental indicator. Specifically, a factor combination is obtained to optimize the experimental indicator, and all factors are sorted according to their influence on the experimental indicators.

The range analysis used the temperature at the target point 20 μm below skin surface as the experimental indicator. The influence law of each parameter on heat penetration is revealed.

The finding in Table 7 present ${\stackrel{\mathrm{-}}{\text{K}}}_{\text{A3}}\text{>}{\stackrel{\mathrm{-}}{\text{K}}}_{\text{A2}}\text{>}{\stackrel{\mathrm{-}}{\text{K}}}_{\text{A1}}$, ${\stackrel{\mathrm{-}}{\text{K}}}_{\text{B1}}\text{>}{\stackrel{\mathrm{-}}{\text{K}}}_{\text{B2}}\text{>}{\stackrel{\mathrm{-}}{\text{K}}}_{\text{B3}}$, ${\stackrel{\mathrm{-}}{\text{K}}}_{\text{C3}}\text{>}{\stackrel{\mathrm{-}}{\text{K}}}_{\text{C2}}\text{>}{\stackrel{\mathrm{-}}{\text{K}}}_{\text{C1}}$, ${\stackrel{\mathrm{-}}{\text{K}}}_{\text{D3}}\text{>}{\stackrel{\mathrm{-}}{\text{K}}}_{\text{D2}}\text{>}{\stackrel{\mathrm{-}}{\text{K}}}_{\text{D1}}$. It suggests that the combination of optimal parameter levels is A3B1C3D3.

**Table 7:** The range analysis for the temperature at the spot center.

Experimental no	Laser power(A)	Spot radius(B)	Irradiation time(C)	Ambient temperature(D)	Target point 20 μm below skin surface
1	5	1	0.2	10	44.190
2	5	1.5	0.4	25	41.418
3	5	2	0.6	35	40.320
4	7.5	1	0.4	35	54.258
5	7.5	1.5	0.6	10	44.122
6	7.5	2	0.2	25	39.628
7	10	1	0.6	25	63.521
8	10	1.5	0.2	35	44.511
9	10	2	0.4	10	41.048
${\text{K}}_{\text{n1}}$	41.98	53.99	42.78	43.12	
${\text{K}}_{\text{n2}}$	46	43.35	45.57	48.19	
${\text{K}}_{\text{n3}}$	49.69	40.33	49.32	46.36	
${\stackrel{\mathrm{-}}{\text{K}}}_{\text{n1}}$	13.99	18.00	14.26	14.37	
${\stackrel{\mathrm{-}}{\text{K}}}_{\text{n2}}$	15.33	14.45	15.19	16.06	
${\stackrel{\mathrm{-}}{\text{K}}}_{\text{n3}}$	16.56	13.44	16.44	15.45	
Optimal levels	A3	B1	C3	D3	
${\text{R}}_{\text{n}}$	7.22	13.66	6.54	5.07	

The ${\text{R}}_{\text{n}}$ values of the parameters are sorted from largest to smallest as the spot radius, laser power, irradiation time, and ambient temperature. The parameter that has the most significant influence on the heat penetration is the spot radius, the second is the laser power, and the smallest is the ambient temperature.

### Analysis of orthogonal experimental result using the variance analysis

Due to the influence of various factors, the data obtained by the orthogonal experiment exhibits fluctuations. The factors that cause fluctuations can be divided into uncontrollable random factors and controllable changed factors. The range analysis cannot distinguish between two types of factors. The variance analysis identifies the factors that have a significant impact on the experimental indicators based on the variance of the observed variables. It gives accurate quantitative estimation, as listed in Table 8.

**Table 8:** The temperature variance analysis.

Variance source	${\text{f}}_{\text{i}}$	F	Significance
Laser Power	2	0.65	**
Spot Radius	2	4.78	*****
Irradiation time	2	0.44	*
Ambient temperature	2	0.26	*

The experimental findings suggest that the most influential factor on the temperature at the target point 20 μm below skin surface is the spot radius, the second is laser power, the third is the irradiation time, and the smallest is the ambient temperature.

#### RBNN training

The results of the error analysis of the developed approximate model are shown in Table 9 below.

**Table 9:** The error analysis results of the surrogate model.

option	RE (%)	$R^{2}$
HPM	[− 0.308, 0.852]	0.9991
ST	[− 0.297, 0.340]	0.9998

**Figure 10:**
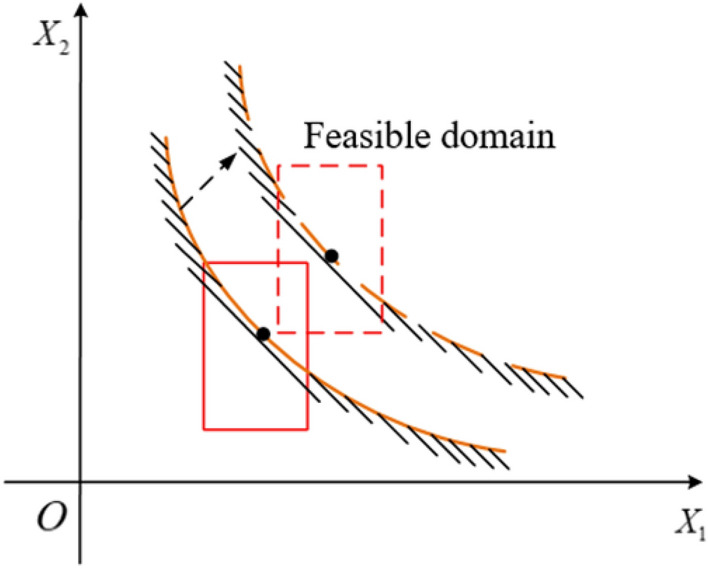
Schematic diagram of uncertainty optimization design.

The results of the established approximation model errors show that the model established by RBNN has sufficient accuracy in medical practice for the penetration of skin temperature during laser thermal pain treatment (Fig. 10).

#### Uncertainty analysis of target parameters

Figure 5 shows the parameter uncertainty variables and their design ranges. The design ranges and interval radii for the four operating parameters of laser power, spot radius, irradiation time, and ambient temperature are included, as shown in Table 10 below.

**Table 10:** The value range and interval radius of moxibustion parameters.

Parameter name	Value range	Interval radius
Laser power (W)	[5, 10]	0.25
Spot radius (mm)	[1, 2]	0.05
irradiation time (s)	[0.2, 0.6]	0.02
ambient temperature (°C)	[10, 35]	1.25

As shown in Table 11, sequential quadratic programming (SQP) was used to calculate the response intervals for the moxibustion indicators based on the RBNN model created.

**Table 11:** The objective function value interval of the mild moxibustion.

Index parameters	Value range
HPM (°C)	[39.0, 58.1]
ST (°C)	[39.2, 59.3]

Table 11 indicates that the skin surface temperature could exceed 52 °C due to the uncertainty of the operating parameters. The actual variation range of the design conditions may cause significant fluctuations in ST, resulting in the failure to achieve the desired therapeutic effect of laser thermal pain stimulation. Figure 4 demonstrates the importance of setting constraints under the uncertainty of the operating parameters. The design conditions are proposed as design points with determined values in the feasible region and away from the failure region. However, due to the influence of the uncertainty of the working parameters, the design condition is a changing region rather than a fixed point. As seen in the figure, a part of the variable domain of the design condition during the treatment is located in the unreliable region, indicating the uncertainty of the treatment effect when the parameters are uncertain. This paper quantifies these uncertainties as interval variables, and their variation domain parameters form a multidimensional box.

To ensure a reliable design, it is essential to ensure that the entire variation domain of the design conditions is located in the feasible region. Therefore, the designs created using the interval uncertainty design method can be reasonably assured of their reliability. Interval optimization can be used as an optimization method for laser thermal pain stimulation, which can improve the effectiveness of the treatment.

#### Nonlinear interval optimization solution

By introducing RPDIs, classical optimization algorithms can be used to solve optimization problems with uncertain intervals. Optimal solutions are computed for different RPDIs.

**Figure 11:**
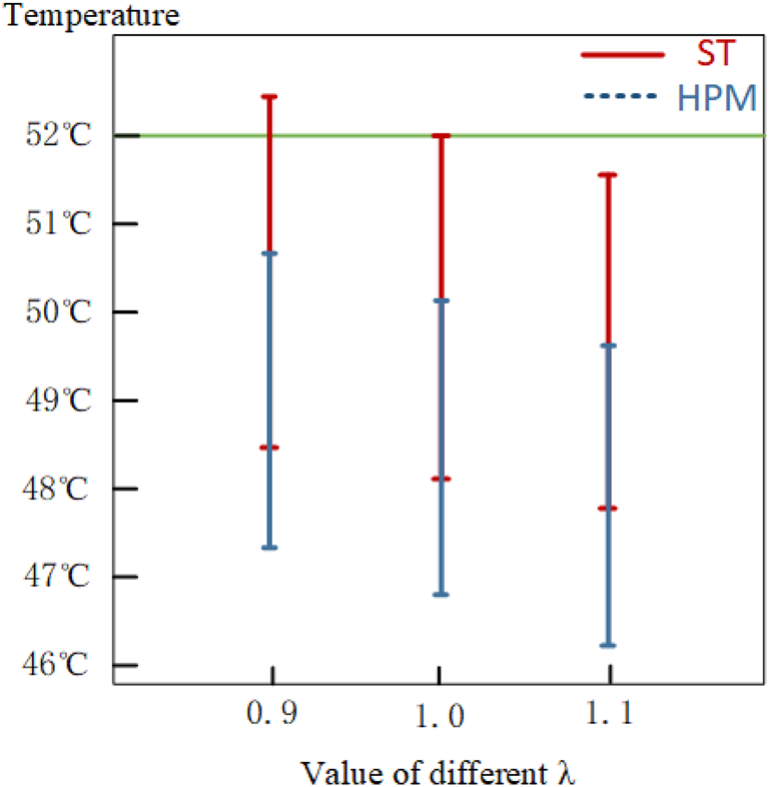
Optimization results under different RPDI.

The study demonstrated that the upper limit of the skin surface temperature interval exceeded the allowable value of 52 °C only when λ was 0.9, but when λ was greater than 1, the upper limit of the skin surface temperature interval was within the allowable limit. The research investigated the impact of different RPDI on thermal penetration and surface temperature intervals to identify the optimal solution for the temperature interval. The findings, illustrated in Table 12 and Fig. 11, indicate that higher RPDI values corresponded to lower thermal penetration and skin surface temperature, implying that an increase in reliability during treatment decreased its efficacy. The results also showed that when the RPDI was greater than or equal to 1, the lowest temperature in the thermal penetration interval was greater than the nociceptive threshold temperature of 43 °C for nociceptive receptors, and the highest temperature in the skin surface temperature interval was less than the damage threshold temperature of 52 °C for skin tissue, thus ensuring reliable laser thermal pain treatment. However, when the RPDI was 0.9, the maximum temperature in the skin surface interval exceeded 52 °C, which could cause damage to the skin tissue.

**Table 12:** The optimal solutions under different RPDIs.

$\lambda value$	0.9	1.0	1.1
ST(°C)	[48.44, 52.39]	[48.07, 52.00]	[47.79, 51.62]
HPM(°C)	[47.17, 50.82]	[46.76, 50.40]	[46.12, 49.71]

## Conclusions

Most previous studies on skin tissue temperature distribution in laser thermal pain treatment have been conducted under deterministic conditions, which cannot ensure stable and reliable treatment outcomes. To accurately assess the effect of operating parameters on target parameters, interval uncertainty analysis and optimization of uncertain parameters were performed to achieve optimal treatment results. First, a simulation and mathematical model of laser thermal pain treatment was established, and the constraint function and objective function were determined. Then, a single-factor analysis was conducted to identify parameters with significant effects. An agent model was introduced, and a neural network (RBNN) was used to establish the constraint function and objective function. An interval uncertainty analysis method was also introduced, considering the uncertainty of parameters that can cause unreliability in the temperature of laser thermal pain treatment. A nonlinear interval optimization model was established, and RPDI was introduced to transform the nonlinear uncertain interval optimization into deterministic interval optimization. Finally, a genetic algorithm was used to solve the deterministic optimization problem. With the increase of RPDI, thermal penetration and surface temperature decreased, and the target parameters were completely reliable when λ was greater than or equal to 1. The results of this study demonstrate that the obtained optimal solution not only significantly improves the efficacy of laser thermal pain treatment, but also ensures the stability and reliability of temperature control. By considering interval uncertainty analysis and optimizing uncertain parameters, the treatment outcomes are more robust and consistent. The findings underscore the importance of accounting for parameter uncertainties in order to achieve reliable and effective laser thermal pain treatment. These results have important implications for enhancing the clinical application of this treatment modality and improving patient outcomes.
